# Locally delivered antistaphylococcal lysin exebacase or CF-296 is active in methicillin-resistant *Staphylococcus aureus* implant-associated osteomyelitis

**DOI:** 10.5194/jbji-7-169-2022

**Published:** 2022-07-27

**Authors:** Melissa Karau, Suzannah Schmidt-Malan, Jay Mandrekar, Dario Lehoux, Raymond Schuch, Cara Cassino, Robin Patel

**Affiliations:** 1 Division of Clinical Microbiology, Department of Laboratory Medicine and Pathology, Mayo Clinic, Rochester, MN, USA; 2 Division of Biomedical Statistics and Informatics, Department of Health Sciences Research, Mayo Clinic, Rochester, MN, USA; 3 ContraFect Corporation, Yonkers, NY, USA; 4 Division of Public Health, Infectious Diseases, and Occupational Medicine, Department of Medicine, Mayo Clinic, Rochester, MN, USA

## Abstract

**Introduction**: *Staphylococcus aureus* is the most common cause of orthopedic infections and can be challenging to treat, especially in the presence of a foreign body. The antistaphylococcal lysins exebacase and CF-296 have rapid bactericidal activity, a low propensity for resistance development, and synergize with some antibiotics. **Methods**: Rabbit implant-associated osteomyelitis was induced by drilling into the medial tibia followed by locally delivering exebacase, CF-296, or lysin carrier. A titanium screw colonized with methicillin-resistant *S. aureus* (MRSA) IDRL-6169 was inserted. Intravenous daptomycin or saline was administered and continued daily for 4 d. On day 5, rabbits were euthanized, and the tibiae and implants were collected for culture. Results were reported as log ${}_{10}$ colony forming units (cfu) per gram of bone or log ${}_{10}$ cfu per implant, and comparisons among the six groups were performed using the Wilcoxon rank sum test. **Results**: Based on implant and bone cultures, all treatments resulted in significantly lower bacterial counts than those of controls ( P≤0.0025 ). Exebacase alone or with daptomycin as well as CF-296 with daptomycin were more active than daptomycin alone ( P≤0.0098 ) or CF-296 alone ( P≤0.0154 ) based on implant cultures. CF-296 with daptomycin was more active than either CF-296 alone ( P=0.0040 ) or daptomycin alone ( P=0.0098 ) based on bone cultures. **Conclusion**: Local delivery of either exebacase or CF-296 offers a promising complement to conventional antibiotics in implant-associated infections.

## 1 Introduction

*Staphylococcus aureus* is the most common bacterium recovered in orthopedic infections (De Araujo et al., 2021; Urish and Cassat, 2020). *S. aureus* forms biofilms on implants and bone and can exist as small colony variants (SCVs) and persister cells; *S. aureus* can enter and survive in cells such as fibroblasts and osteoblasts, rendering it difficult for the immune system and antibiotics to come in to contact with the bacteria (Alder et al., 2020; Muthukrishnan et al., 2019; Nasser et al., 2020; Waters et al., 2016). Thus, there is an urgent, unmet need for improved antimicrobials that are fast acting and target biofilms to help manage associated infections.

Lysins are cell wall hydrolytic enzymes being developed for therapeutic use as direct lytic agents. They are recombinantly produced as purified proteins from genetic material derived from bacteriophages (Fischetti, 2008). Exebacase (previously known as CF-301) and CF-296 are antistaphylococcal lysins with activity against *S. aureus*. Exebacase has a similar domain arrangement to most phage lysins with cell wall-cleaving and -binding properties at the N-terminal and C-terminal domains, respectively (Schuch et al., 2014). CF-296 is an engineered variant of exebacase, developed to potentially maintain longer anti-biofilm activity and be administered in repeated and/or higher doses than exebacase. In vitro studies show activity of exebacase against a range of *S. aureus* strains with rapid bactericidal activity, low propensity for resistance development, enhancement in the presence of human serum components, synergy with several antibiotics, and lack of selection for SCVs or persister cells (Indiani et al., 2019; Kebriaei et al., 2021; Oh et al., 2019; Schuch et al., 2017, 2014; Swift et al., 2021; Watson et al., 2020). Animal studies have shown enhanced activity of exebacase and CF-296 in combination with conventional antibiotics in different models of biofilm-associated *S. aureus* infections (Asempa et al., 2019; Karau et al., 2021, 2019; Schuch et al., 2014; Shah et al., 2020; Swift et al., 2021). Recently, a phase 2 clinical trial (NCT03163446) was completed testing the efficacy and safety of exebacase as an addition to standard antibiotics for treatment of *S. aureus* bacteremia and endocarditis, showing improved clinical outcomes in the methicillin-resistant *S. aureus* (MRSA) subset (Fowler et al., 2020). Currently, exebacase is being studied in a phase 3 clinical trial (NCT04160468) for treatment of *S. aureus* bacteremia, including right-sided endocarditis (Clinicaltrials.Gov, 2021).

The referenced in vivo studies delivered lysin systemically; locally delivered lysin has been incompletely studied. Here, using a rabbit model of implant-associated osteomyelitis with bone-implanted screws colonized with MRSA, we tested the activity of exebacase or CF-296 delivered locally with and without systemic daptomycin therapy.

## 2 Methods

### 2.1 Materials

Exebacase and CF-296 were supplied as 10.64 and 10.16 mg mL ${}^{-1}$ , respectively, in a liquid carrier solution containing proprietary components (ContraFect Corporation, Yonkers, NY). Daptomycin (Xellia Pharmaceuticals USA Inc., North Wales, PA) was supplied as 500 mg of powder and was reconstituted in 10 mL of sterile 0.9 % sodium chloride. Implants were 1.5 mm ×7 mm titanium cortex screws (DePuy Synthes, Monument, CO). The study strain was MRSA IDRL-6169, an isolate recovered at the Mayo Clinic from a periprosthetic hip infection, which had minimum inhibitory concentrations (MICs) for daptomycin, exebacase, and CF-296 of 0.5 $µg\;{\mathrm{mL}}^{-1}$ .

### 2.2 Implant colonization

**Figure 1:**
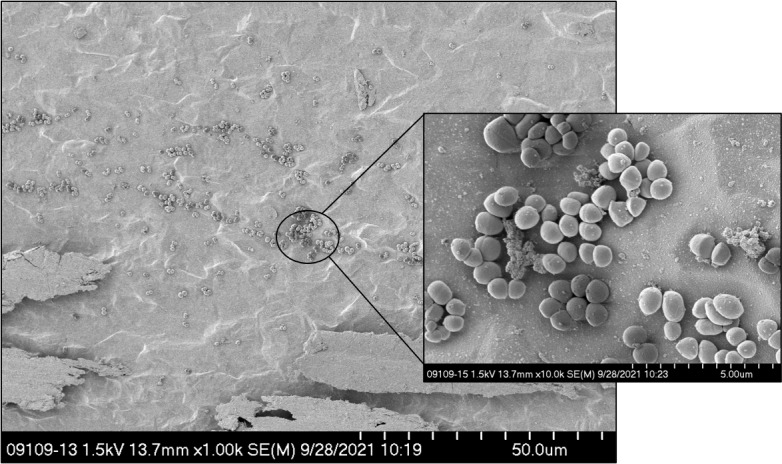
Scanning electron microscopy of the MRSA-seeded implant.

To colonize individual implants with MRSA, 37 µL of a 10 ${}^{4}$ colony forming units (cfu) per milliliter suspension of MRSA IDRL-6169 in trypticase soy broth (TSB) was added to microcentrifuge tubes containing individual titanium screws and placed on an orbital shaker at 37 ${}^{\circ }$ C for ∼16 h. On each surgical day, pre- and post-surgery, one screw was quantitatively cultured to determine representative log ${}_{10}$ cfu per implant. Figure 1 shows a representative image of the seeded implant.

### 2.3 Biofilm time–kill curve

Time–kill studies were performed to confirm lysin activity against seeded implants. Screws were seeded with bacteria and placed into either 0.1 mL of lysin carrier, exebacase, or CF-296 for 1, 2, 4, 8, 12, or 24 h at 37 ${}^{\circ }$ C. Three screws per time point for each treatment were quantitatively cultured. Briefly, screws were removed from treatment, dipped into saline, placed into 1 mL saline, vortexed and sonicated, and quantitatively cultured. Results were reported as log ${}_{10}$ cfu per screw.

**Figure 2:**
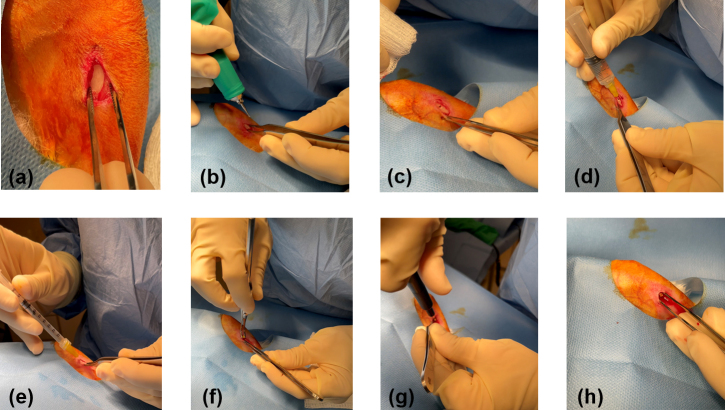
Surgical procedure: an incision was made over the left medial tibia, and the smooth flat portion was exposed **(a)**; using a micro drill, a hole was created through the cortical bone into the medullary cavity **(b, c)**; a total of 2 mL of water **(d)** followed by 0.6 mL of lysin or lysin carrier **(e)** was injected; and a seeded implant was inserted **(f)**, tightened with a screwdriver **(g)**, and confirmed to be secure **(h)**.

### 2.4 Surgical methods

**Figure 3:**
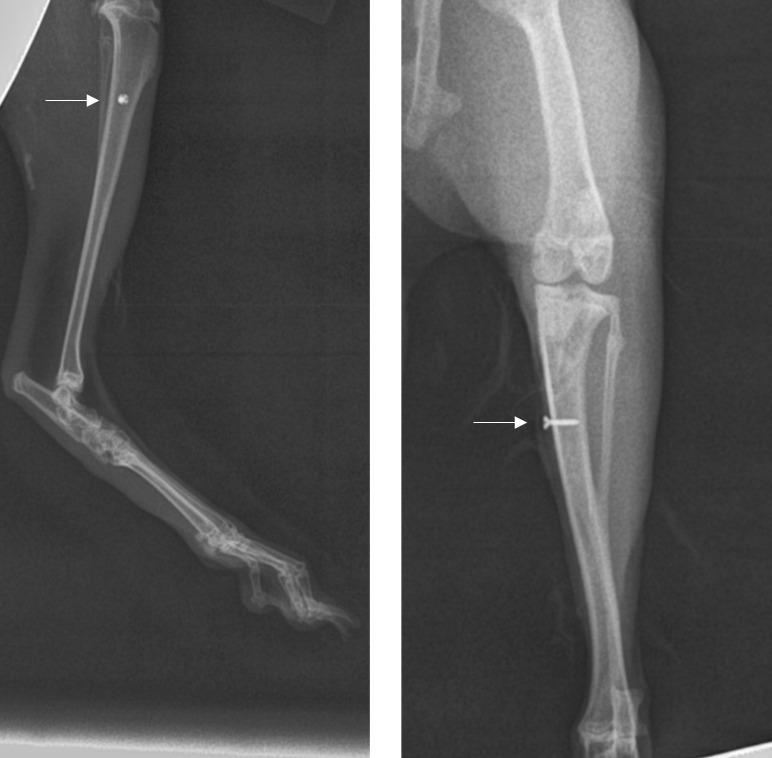
Medial and supine X-rays of the left rabbit tibia to show implant placement (arrow).

A total of 60 New Zealand male and female rabbits (10–12 weeks old, 2.5–3.0 kg) were studied. All animals tolerated procedures well with no unintentional loss of life throughout the study. Each surgical day, rabbits were randomly placed into one of six treatments groups (systemic/local) with 10 rabbits per group: saline/lysin carrier, daptomycin/lysin carrier, saline/exebacase, daptomycin/exebacase, saline/CF-296, or daptomycin/CF-296. Rabbits were administered anesthesia and analgesia. A 1.5 cm incision was made on the proximal portion of the medial left tibia. The fascia and muscle were cleared to expose a smooth flat portion of the tibia (Fig. 2a). Using micro drill with a 1.4 mm round burr, a hole was punched through the cortical bone into the medullary cavity (Fig. 2b, c). To remove bone fragments, 2 mL of sterile water was flushed into the tibia (Fig. 2d) followed by 0.6 mL of exebacase ( ∼6.4 mg equivalent), CF-296 ( ∼6.1 mg equivalent), or lysin carrier (Fig. 2e). Concurrently, systemic treatment of either daptomycin (6 mg kg ${}^{-1}$ ) or saline was delivered intravenously. An implant with ∼ 5.24 log ${}_{10}$ cfu per implant was placed into the hole (Fig. 2f, g, h), and the site was closed. Figure 3 is a representative X-ray of the implant.

### 2.5 Post-operative management

Daptomycin (6 mg kg ${}^{-1}$ ) or saline was administered intravenously daily for 3 d. A rabbit dose of 12 mg kg ${}^{-1}$ of daptomycin results in a similar area under the plasma concentration curve to a human dosing of 6 mg kg ${}^{-1}$ (Chambers et al., 2009); however, a daptomycin dose of 6 mg kg ${}^{-1}$ was selected to assess the effects of combination activity. On day 5, rabbits were euthanized, and the tibiae and implants were aseptically collected and cultured separately.

Whole tibiae were cryopulverized with a Mixer Mill MM 400 (Retsch, Newtown, PA). The resultant bone powder was suspended in 10 mL saline, and the implants were placed in 1 mL saline. Tubes were vortexed and sonicated for 5 min and quantitatively cultured. Results were reported as log ${}_{10}$ cfu g ${}^{-1}$ of bone or implant. MICs were performed on recovered MRSA to assess for the emergence of resistance.

### 2.6 Statistical analyses

Descriptive summaries are reported as medians. A sample size of 10 per group was chosen based on an 80 % power to detect a difference of 1.3 standard deviations or larger based on a two-sided two-sample test at an α level of 0.05. Comparisons among the six groups were first performed using the Kruskal–Wallis test. Due to statistically significant differences between the groups, further comparisons between the groups were performed in a pairwise manner using the Wilcoxon rank sum test. Nonparametric tests were used due to the small sample size and non-normally distributed data. No adjustment was made for multiple comparisons. All tests were two sided, with P values less than 0.05 considered statistically significant. Analysis was performed using SAS software version 9.4 (SAS Institute Inc, Cary, NC).

**Figure 4:**
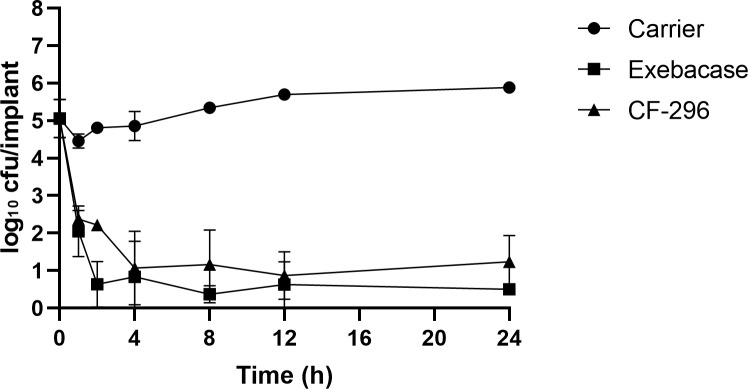
Implant time–kill study. Titanium screws grown overnight with MRSA IDRL-6169 were placed in 0.1 mL of lysin carrier, exebacase (10.64 mg mL ${}^{-1}$ ), or CF-296 (10.16 mg mL ${}^{-1}$ ). After 1, 2, 4, 8, 12, or 24 h, three screws each were processed for quantitative culture and results were reported as log ${}_{10}$ cfu per implant.

**Figure 5:**
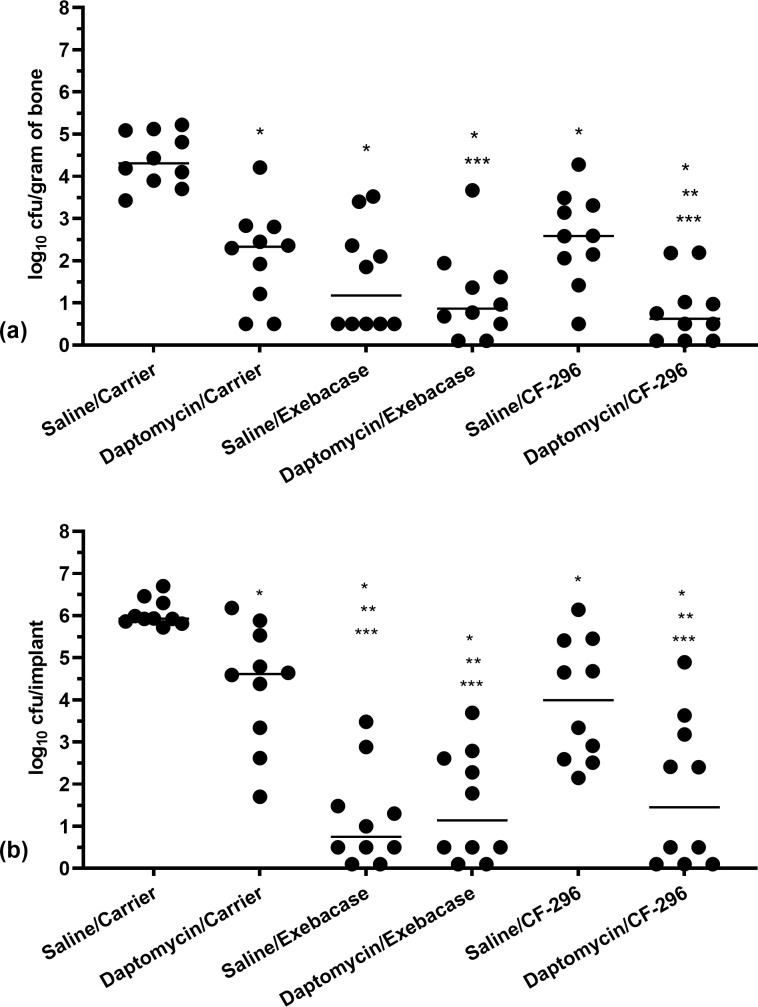
Quantities of MRSA recovered from bones **(a)** and implants **(b)** on day 5. Dots represent values from individual animals; n=10 per group; horizontal lines represent median values. Asterisks indicate significant reductions compared to * saline/carrier ( P≤0.0025 ), ** daptomycin/carrier ( P≤0.0098 ), or *** saline/CF-296 ( P≤0.0154 ). (Note that slashes depict the treatment grouping, with the top being the systemic treatment given daily and the bottom being the local treatment administered at the time of surgery.)

## 3 Results

### 3.1 In vitro results

In an in vitro biofilm time–kill experiment (Fig. 4), the mean bacterial load prior to treatment was 5.06 log ${}_{10}$ cfu per implant. Compared with the lysin carrier, there was a mean reduction of 2.41 and 2.08 log ${}_{10}$ cfu per implant after 1 h and 5.38 and 4.65 log ${}_{10}$ cfu per implant after 24 h for exebacase and CF-296, respectively, indicating bactericidal activity (Pankey and Sabath, 2004).

### 3.2 In vivo results

Results of the bone and screw cultures are shown in Fig. 5a and b, respectively. All treatments (systemic intravenous treatment/local delivery) studied were active compared with controls (saline/lysin carrier), whether based on bone or implant cultures ( P≤0.0025 ). For bone and implant cultures, there were >3 and >4 log ${}_{10}$ cfu reductions, respectively, for saline/exebacase, daptomycin/exebacase, and daptomycin/CF-296, compared with saline/carrier. Compared with bones of daptomycin/carrier, there were 1.15, 1.46, and 1.70 log ${}_{10}$ cfu reductions in bones of animals receiving saline/exebacase, daptomycin/exebacase, and daptomycin/CF-296, respectively. In addition, bacterial densities in bones were less than 1 log ${}_{10}$ cfu in 50 %, 60 %, and 70 % of animals in those same groups, respectively. Daptomycin/CF-296 resulted in significant reductions in *S. aureus* recovered from bones and implants compared with daptomycin/carrier ( P=0.0098 and P=0.0064 , respectively) or CF-296 alone ( P=0.0040 and P=0.0154 , respectively). Bacterial densities on implants were reduced by 3.87 ( P=0.007 ), 3.48 ( P=0.0015 ), and 3.17 ( P=0.0064 ) log ${}_{10}$ cfu for animals treated with saline/exebacase, daptomycin/exebacase, and daptomycin/CF-296, respectively, compared with daptomycin/carrier-treated animals. In these respective groups, 60 %, 50 %, and 50 % of the animals had ≤1 log ${}_{10}$ cfu recovered from their implant. Exebacase alone resulted in greater cfu reductions on implants than locally delivered CF-296 alone ( P=0.0015 ); there was no difference between the two lysins' activity when delivered locally in conjunction with systemic daptomycin, whether based on bone or implant cultures. Overall, locally delivered exebacase alone or with systemic daptomycin and locally delivered CF-296 with systemically delivered daptomycin showed the most activity. No emergence of resistance was found to daptomycin nor to either lysin.

## 4 Discussion

Local delivery of antimicrobials directly to the site of infection in addition to systemic therapy has been an area of interest with the potential to overcome some hurdles associated with traditional therapies (e.g., low biofilm activity, tissue penetration, and intracellular activity). Applying a treatment locally to an affected area allows immediate, direct contact with the infecting agent(s), compared with delayed time of delivery and variable low concentrations of available drug with systemic dosing. Over the past 20 years, vancomycin powder has been used in spine surgeries to prevent surgical site infections (Dodson et al., 2019). It is also used in fractures and traumatic injuries (O'Toole et al., 2021). More recently it has been used in primary joint arthroplasties and revisions, albeit with variable results (Movassaghi et al., 2022; Saidahmed et al., 2021). We recently showed vancomycin powder to be active against *Staphylococcus epidermidis* biofilms on spinal implants in vivo (Karau et al., 2020).

Exebacase has shown excellent in vitro activity against *S. aureus* which hypothetically indicates that activity could be leveraged with locally delivered lysin. When planktonic cells were exposed to exebacase, Schuch et al. (2014) observed the loss of cytoplasmic components within 15 s when viewed by electron microscopy, with >3 logs of killing in 30 min as assessed by time–kill curves. There was faster binding of daptomycin or vancomycin to bacterial cell walls in the presence of exebacase (Schuch et al., 2014). Exebacase has been shown to disrupt *S. aureus* biofilms grown on several surface types. Exebacase disrupted biofilms grown on catheters within 1 h and killed bacteria within 6 h; exebacase has also shown activity against SCVs and persister cells (Schuch et al., 2017). Additionally, synergy has been observed in time–kill assays with ≥2 log ${}_{10}$ reductions when exebacase was used in addition to either daptomycin or vancomycin, compared with exebacase or antibiotic alone (Schuch et al., 2014; Watson et al., 2020).

The current model used orthopedic screws colonized with MRSA and treated with locally delivered lysin; results were reproducible, and rabbits tolerated the procedures well. Given the limited activity of many traditional antibiotics against implant-associated infections, these results indicate that locally delivered lysins can reduce bacterial loads in vivo. When used with systemic daptomycin, there were marked reductions in bacterial loads. Bactericidal activity was shown based on both implant and bone cultures, with a greater than 3 log ${}_{10}$ reduction in bacterial counts with systemic daptomycin combined with locally delivered lysin, with three animals having no detectable bacteria in either sample type studied.

In a mouse periprosthetic knee *S. aureus* infection model incorporating debridement and implant retention (DAIR), Sosa et al. (2020) recently showed that the intra-articular and systemic administration of exebacase in combination with vancomycin reduced bacterial loads on implants and in surrounding tissues, although not in bone, when compared with vancomycin alone. There was no evaluation of exclusive local delivery, as was done herein. In a rat model of implant-associated osteomyelitis using a tibial screw implant, a CRISPR-Cas9 modified phage was administered into the bone with and without fosfomycin administered locally in an alginate hydrogel and compared to the alginate hydrogel or fosfomycin alone after resection the implant (Cobb et al., 2019). After 1 d, there was a reduction in MRSA in soft tissues compared with no treatment, but there was no difference in activity between fosfomycin, phage, nor the combination thereof. Although combination therapy was not more effective than the other treatments studied, fosfomycin was more active than alginate hydrogel or phage alone based on bone cultures.

In France, where exebacase has been administered on a compassionate-use basis into knees of elderly patients with recurrent methicillin-resistant *S. epidermidis* periprosthetic joint infection undergoing DAIR who had limited therapeutic options (Ferry et al., 2021), two of the four patients studied had favorable outcomes after 1 year, supporting our findings and suggesting that local administration of lysins should be further pursued.

These studies, along with ours, suggest that the local administration of lysins in addition to systemic antibiotics could be an option in treating implant-associated *S. aureus* infection. In our study, using such an approach, reductions in MRSA in bone and on implants in all treatment groups were shown compared with control animals. Moreover, a combination of CF-296 and daptomycin was more active than either daptomycin or CF-296 alone. We demonstrated excellent activity against MRSA with exebacase, a combination of exebacase and daptomycin, and a combination of CF-296 and daptomycin, with all being more active than daptomycin alone on implants.

There are several limitations to this study. First, only one strain of MRSA, one concentration of lysin, and one type of antibiotic were studied. Second, daptomycin was intentionally underdosed with reference to typical human dosing, to address activity in addition to the lysins; further work with varying concentrations should be performed to address synergy. Third, this novel model used colonized screws treated at time of insertion, a somewhat contrived scenario designed to allow assessment of the activity of locally delivered lysins against colonizing bacteria in vivo. Finally, there was less activity in bone than on implant surfaces; a possible explanation for the lesser degree of activity in bone could be the inability of lysins to reach bacteria residing within bone matrices and in cells. The combination of a bone-penetrating systemic antibiotic and local delivery of a fast-acting lysin to target biofilms on implants may provide a treatment option for these complicated infections.

In summary, an acute MRSA implant-associated osteomyelitis model was used to test local delivery of antistaphylococcal lysin in additional to systemic delivery of daptomycin. All treatment groups had significantly reduced amounts of MRSA recovered from both bone and implants, with the most active treatments being locally delivered exebacase alone and either lysin, exebacase, or CF-296, delivered locally in addition to systemic daptomycin. There was no difference between the activity of the two lysins delivered locally when administered with systemic daptomycin. Lysins administered locally, in combination with traditional therapies, may offer a potential strategy for combatting *S. aureus* implant-associated infections.

## Data Availability

Data are available upon request.
